# The dynamic changes of interferon lambdas related genes and proteins in JAK/STAT pathway in both acute and chronic HIV-1 infected patients

**DOI:** 10.1186/s12981-017-0158-7

**Published:** 2017-06-17

**Authors:** Guoxian Zhao, Lifeng Liu, Bin Su, Tong Zhang, Peng Chen, Wei Li, Hao Wu

**Affiliations:** 1 Center for Infectious Diseases, Beijing You’an Hospital, Capital Medical University, Beijing, 100069 China; 2 Beijing Key Laboratory for HIV/AIDS Research, Beijing, 100069 China; 3 Center of Interventional Oncology and Liver Diseases, Beijing You’an Hospital, Capital Medical University, Beijing, 100069 China

**Keywords:** Acute HIV-1 infection, IFN-lambdas, Signaling pathway, STAT1, Mx1

## Abstract

**Background:**

Host immune responses during acute HIV-1 infection can influence the viral setpoint, which is a predictor of disease progression. Interferon (IFN)-lambdas are newly classified type III interferons, which use JAK-STAT pathway. Currently, the dynamics of IFN-lambdas related genes and proteins expression in the signaling pathway have not been well elaborated, especially in acute HIV-1-infected patients.

**Objectives:**

To evaluate the dynamic changes of IFN-lambdas related genes and proteins in JAK/STAT pathway in acute HIV-1-infected patients, and analyze their correlation with CD4 T cell counts and HIV-1 viral loads.

**Study design:**

Real-time PCR and flow cytometry methods were used to evaluate the dynamic changes of IFN-lambdas related genes and proteins in JAK/STAT pathway in both acute and chronic HIV-1-infected patients.

**Results:**

The IFN-alpha receptors (R), IFN-gamma R, IFN-lambdas R and STAT1 mRNA and protein levels increased in acute HIV-1-infected patients (*p* < 0.01), in addition, Mx1 mRNA levels in acute HIV-1-infected patients are higher than those in HIV-negative subjects. IFN-lambdas R and IFN-alpha R mRNA levels are inversely correlated with CD4^+^ T-cell counts, but are positively correlated with viral loads.

**Conclusions:**

The dynamic changes of IFNs related genes in JAK-STAT pathway in acute HIV-1 infection will deepen our understanding of the roles of IFN-lambdas in HIV pathogenesis.

## Background

Host immune responses during acute HIV-1 infection can influence the establishment of the viral setpoint, which is a predictor of disease progression, therefore, it is of importance to characterize the immune responses during acute HIV-1 infection. Viral infection can induce the production of interferons, which use JAK-STAT pathway [1]. Interferons (IFN)-lambdas are newly classified type III interferons [2, 3], until now, the dynamics of IFN-lambdas related genes and proteins expression in the signaling pathway have not been well elaborated, especially in acute HIV-1-infected patients.

IFN-α and IFN-β are main type I interferons, 12 closely related genes encode IFN-α. IFN-γ is the sole type II interferon. In 2003, type III IFNs which include three IFN-lambdas genes were newly identified by two independent groups. The receptor (R) of the type III IFNs is composed of a short chain (IL-10R2) and a long chain (IL28AR), IL28AR is utilized only by IFN-λ [2, 3]. IL28AR was shown to be expressed on some blood cells including B cells, T cells, dendritic cells (DCs) and macrophages [4]. Type III IFNs trigger responses through the JAK/STAT pathway, after binding to their receptors, JAK and STAT proteins are activated. There are six types of STAT proteins in the pathway: STAT1, STAT2, STAT3, STAT4, STAT5a, STAT5b, STAT6. Activated STAT1, STAT2, and interferon regulatory factor 9 (IRF-9) form a complex called IFN-stimulated gene factor 3 (ISGF3), once assembled, ISGF3 is then translocated into nucleus where it binds to the IFN-stimulated response elements (ISRE), ISRE is located in the promoter of numerous IFN stimulated genes (ISGs). Some ISG-encode proteins mediate a variety of antiviral activities, such as myxovirus resistance protein A (MxA) and 2′,5′-oligoadenylate synthetase (OAS) [5].

IFNs are generally considered as cytokines with strong antiviral activities. The dynamics of type I and II IFNs in acute and chronic HIV-1-infected patients have been reported by previous studies. IFN-alpha is transiently expressed in acute HIV-1 infection (AHI), type I IFNs consist of several subtypes, only IFN-alpha is significantly elevated in chronic HIV-1 infection [6, 7]. IFN-gamma is detected as early as the acute phase and continually detected throughout the course of infection. In chronic, clinical progression or non-progression patients, IFN-gamma persistently elevated [8, 9]. IFN-lambdas can induce type-1 IFN-like antiviral response and blocks HCV infection in human primary hepatocyte and HUH7 cell lines [10]. IFN-lambdas can induce IFN-α/β-like antiviral response and inhibition of HBV replication in murine heptocyte cell lines [11]. IFN-lambda3 can inhibit HIV infection of macrophage through the JAK-STAT pathway and induce antiviral state in culture primary T-cells. When macrophages were pretreated once with IFN-lambdas, a nearly complete inhibition of HIV-1 replication in macrophages was observed [12].

Based on the ongoing burden of HIV infection among men who have sex with men (MSM) in China, we established an open Beijing Primo Cohort study in 2007 to identify acute HIV-1 infections [13]. This cohort study, with carefully estimated dates of seroconversion, has afforded us the great opportunity to investigate the dynamic changes of IFN-lambdas related genes and proteins expression in JAK/STAT pathway in acute HIV-1-infected patients. Moreover, phenotypic and functional perturbations of monocyte subsets in acute and chronic HIV-1 infected patients were characterized using this Beijing Primo Cohort [14]. Until now, the dynamics of IFN-lambdas related genes and proteins expression in the signaling pathway have not been well elaborated, especially in acute HIV-1-infected patients.

In the study, real-time PCR and flow cytometry methods were used to evaluate the dynamic changes of IFN-lambdas related genes and proteins in JAK/STAT pathway in acute HIV-1-infected patients, and then we analyzed their correlation with CD4^+^ T-cell counts and HIV-1 viral loads.

## Objectives

The aim of this study was to evaluate the dynamic changes of IFN-lambdas related genes and proteins in JAK/STAT pathway in AHI patients, and evaluate their correlation with CD4^+^ T cell counts and viral loads.

## Study design

### Study subjects

Twenty MSM with acute HIV-1 infection (AHI) were enrolled from an open Beijing Primo Cohort study in 2007 to identify acute HIV-1 infections. The progression of early HIV-1 infection can be depicted as six discrete stages as proposed by Fiebig et al. [15, 16]. These 20 AHI patients were at Fiebig stage III–V. Twenty-four HIV-1-infected treatment-naïve patients were randomly enrolled from HIV/AIDS clinic of Beijing You’an Hospital, these cART-naïve patients with chronic HIV infection (CHI) were diagnosed at least 1.2 years (median 1.8 years, range from 1.2 to 2.3 years) before enrollment. The patients who have accepted antiretroviral therapy (ART) or were the intravenous drug users or co-infected with HBV/HCV were excluded from this study. Twenty-two male MSM HIV-negative controls (HC) were included as controls, the ages of all groups were matched.

The protocol for this study was approved by the Beijing You’an Hospital Ethics Committee and written informed consent was provided according to the declaration of Helsinki. The guidelines for Human Experimentation (PR China) were followed throughout. Upon admission, all patients provided written informed consent for their information, clinical samples (blood and plasma) were stored and used for research. The methods were carried out in accordance with approved guidelines and regulations. 5800 men who have sex with men (MSM) were screened by our team and were followed up every 2–3 months. Once acute HIV-1-infected cases were identified, blood samples were collected, and then PBMCs were isolated and cryopreserved.

### RNA extraction and quantitative RT-PCR

Viral RNA was purified from peripheral blood mononuclear cell (PBMC) samples using the QIAamp Viral RNA Mini Kit (Qiagen, Duesseldorf, Germany) according to the manufacturer’s protocol. Total cDNA was synthesized by reverse transcription using First Strand cDNA Synthesis Kit (Invitrogen, USA) with random primers. The real-time RT-PCR for the quantification of IFN, IFN R, β-actin and other genes were performed with the Power SYBR^®^ Green PCR Master Mix (Invitrogen, USA). The primers used in this study were listed in Table 1.

**Table 1:** Nucleotide sequences of the primers used for real-time PCR

Target gene	Accession number	Type	Primer sequence	Productamplicon size (bp)
IFN lambda 1	NM_172140	F R	GTGCTGGTGACTTTGGTGCTA GAGAAGCCTCAGGTCCCAAT	225
IFN alpha R2	NM_207584.2	F R	GTCAGGCCTCTGCCACCTCT GGGTCCTGGAGTGGACTCTTTCT	172
IFN gamma R1	NM_000416.2	F R	GCCAGGGTTGGACAAAAAGA GACTTCCTGCTCGTCTCCATTTA	168
IFN lambda R1	NM_170743.3	F R	CCTCAGTGCCAGAACCATCTACA CCATGAGGGTCTTCCAGATCACA	152
STAT1	NM_007315.3	F R	CCCTTCTGGCTTTGGATTGAA CTTCCCGGGAGCTCTCACTGA	172
STAT2	NM_198332.1	F R	GAGAGCCCTCCTGGCAAGTTA GACGATTCACTGAAGCGCAGT	185
STAT3	NM_003150.3	F R	TTGCCAGTTGTGGTGATCTCC GCTGCTCGATGCTCAGTCCTC	199
STAT4	NM_003151.3	F R	TCAGGAAGGGCAAGCACTGGT GGAAGGGATGGGTAGCCAGGA	150
STAT5A	NM_003152.3	F R	GGATCCTGGTTGACGCCATGT GCTTGGATCCTCAGGCTCTCC	122
STAT5B	NM_012448.3	F R	GCTGGAAGCCTTGCTGATGCC CTGAGCTTGGATCCTCAGGCTCT	114
STAT6	NM_003153.4	F R	GCCCACTCACTCCAGAGGACCT GGTGTTGGGGAAAGTCGACAT	121
IRF9	NM_006084.4	F R	GAGCAAGTGGAGAGTGGGCAGTT CCAGACAGCTGGACCTCCTGTGT	189
OAS1	NM_016816.2	F R	CAGTTAAATCGCCGGGGAGAGT GCTGGAGCGAACTCAGTACGAA	101
MX1	NM_002462.4	F R	GGTGGTGGTCCCCAGTAATG CCGGCACTTGACAATCATGT	150
Beta actin	NM_001101.3	F R	GCACGGCATCGTCACCAACT GCTGGGGTGTTGAAGGTCTCA	177

*F* forward primer, *R* reverse primer, *bp* base pair, *STAT* signal transducer and activator of transcription, *IRF9* IFN regulatory factor 9, *OAS1* 2′,5′-oligoadenylate synthetase 1, *MX1* myxovirus resistance 1

The reaction mixture was first denatured at 95 °C for 4 min and then 40 cycles of PCR were performed using the following protocol: 95 °C 30 s; 60 °C 30 s; 72 °C 30 s. The mRNA level of each selected gene was normalized with β-actin to obtain mRNA relative expression level.

The quantification of PCR data was achieved using the comparative Ct method. We calculated the 2^−∆∆Ct^, where ∆∆Ct = ∆Ct_sample_−∆Ct_reference_. The ∆Ct_sample_ was calculated as Ct value for any sample normalized to the endogenous housekeeping gene and ∆Ct_reference_ was the Ct value for the control also normalized to the endogenous housekeeping gene. The mean value of 2^−∆∆Ct^ from control group was considered equal to 1; the fold change over the mean 2^−∆∆Ct^ of controls was calculated for all samples.

### Antibodies and flow cytometry

Antibodies (Abs) used in this study were as follows: mouse phycoerythin (PE)-conjugated anti-human IFN-alpha/beta R2 Ab (clone MMHAR-2, R&D Systems), mouse PE-conjugated anti-human IFN-lambdas R1 Ab (clone 601106, R&D Systems), mouse PE-conjugated anti-human IFN-gamma Receptor 1 (clone GIR-208, Thermo Fisher Scientific), Mouse eFluor^®^ 450-conjugated anti-human STAT1 (KIKSI0803, Thermo Fisher Scientific), Rabbit FITC anti-Human STAT2 (Thermo Fisher Scientific), Mouse PE-Cyanine7-conjugated anti-human STAT3 (clone LUVNKLA, Thermo Fisher Scientific), mouse APC-conjugated anti-human STAT4 (clone 4LURPLE, Thermo Fisher Scientific), mouse FITC-conjugated anti-human STAT5 (clone SRBCZX, Thermo Fisher Scientific), mouse PerCP-eFluor^®^ 710-conjugated anti-human STAT6 (clone CHI2S4N, Thermo Fisher Scientific). All STATs Abs were used in phosphorylated state, phosphorylation of STATs occur in response to IFN, and is essential for normal transcriptional activity of the IFN stimulated gene factor 3 complex. Rabbit Alexa Fluor^®^ 488 conjugated anti-human IRF9(clone EPR3939, Abcam), purified rabbit anti-human MxA Ab (Abcam) purified mouse anti-human OAS1Ab (Abcam), goat DyLight^®^ 488 conjugated anti-mouse IgG (H+L) (Abcam). The isotype control antibodies were purchased from the corresponding company, respectively.

Cryopreserved peripheral blood mononuclear cells (PBMCs) were used, and cells were stained with appropriate target Abs and isotype Abs using conventional surface and/or intracellular staining methods. Cryopreserved PBMCs were thawed in RPMI 1640 medium (Invitrogen, Carlsbad, CA, USA), washed with PBS, and then incubated at room temperature for 20 min with the cells viability marker fixable viability stain 510 (BD Biosciences, San Jose, CA, USA). When intracellular staining was required, cells were first fixed and permeabilized using BD Cytofix/Cytoperm Fixation and Permeabilization Solution (BD biosciences), followed by staining for intracellular proteins. Forward scatter (FSC) and side scatter (SSC) light gating were used to exclude cell debris from the analysis. Forward height and forward area were used to exclude doublet cells, and cell viability were evaluated. All expression analysis was performed by flow cytometry using BD FACSCanto™ II with Diva software (BD, Franklin Lakes, NJ), and the data were analyzed using FlowJo 10.0.7 software (Tree Star, Ashland, OR, USA).

### CD4^+^ T-cell count and viral loads measurement

CD4^+^ T cells were determined by three-color flow cytometry using CD3-APC, CD4-FITC, and CD8-PE monoclonal antibodies (BD Biosciences). Analysis was then carried out by a BD FACSCanto™ II Flow cytometry.

HIV-1 Viral loads tests were done by using an automated real-time PCR-based *m*2000 system (Abbott Molecular Inc. Des Plaines, IL) as manufacturers’ instruction and the sensitivity of detection was 40 copies/ml.

### Statistical analyses

Statistical analysis was performed using Student’s *t*-test or Non parametric tests. All reported *p* values were two-sided and considered significant at *p* < 0.05. The association was evaluated by Spearman’s correlation test. All data were analyzed using SPSS 21.0 statistical software (Chicago, IL, USA).

## Results

### Patients’ characteristics

Twenty MSM with acute HIV infection, twenty-four chronic HIV infection and twenty-two HIV-negative controls were enrolled in the study. Basic information of these patients and 22 controls is presented in Table 2. The age between HIV-1-infected patients and HIV-negative controls is matched. The viral loads of HIV-negative controls are not available. Mean CD4^+^ T cell counts of acute HIV infected patients are lower than those of chronic HIV-1-infected patients and HIV-negative controls (*p* = 0.006).

**Table 2:** Characteristics of HIV-1-infected patients and HIV-negative controls

	Chronic HIV infected patients	Acute HIV infected patients	HIV-negative control	p value
Cases, no	24	20	22	
Age (years)	33.8 (20–45)	33.2 (18–46)	34.6 (20–49)	0.738
HIV-RNA (copies/ml)	9955 (861–16,483)	22,438 (2029–189,695)	NA	NA
CD4 (cells/μl)	538 (476–789)	363 (164–692)	687 (401–1209)	0.0028

Data are presented as the means with ranges

*NA* not available

### The mRNA levels of IFN receptors and related genes during acute and chronic HIV-1 infection

**Fig. 1:**
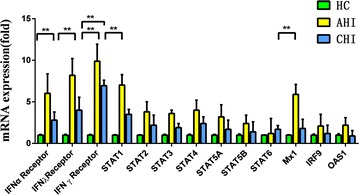
The IFNs related genes of mRNA levels in HIV-negative controls (HC), acute HIV-1-infected (AHI) patients and chronic HIV-1-infected (CHI) patients. Total RNA extracted from PBMC was subjected to the real-time PCR. The data are expressed as mRNA levels relative (fold) to the control (which defined as 1). The results are shown mean ± standard deviation, the *asterisks* denote *p* < 0.01

In the study, the mRNA levels of the following genes were evaluated by real time PCR: IFN-alpha/beta R2, IFN-gamma R1, IFN-lambdas R1, STAT1, STAT2, STAT3, STAT4, STAT5a, STAT5b, STAT6, IRF9, Mx1, OAS1. As is shown in Fig. 1, IFN-alpha R, IFN-gamma R, IFN-lambdas R, STAT1 and Mx1mRNA levels in acute HIV infected patients were significant higher than those in HIV-negative controls (*p* < 0.01). The IFN-alpha R, IFN-gamma R, IFN-lambdas R, STAT1 and Mx1 R mRNA levels of acute infected patients are 6.03, 9.89, 8.20, 7.03 and 5.89 times higher than those of HIV-negative controls accordingly. In addition, the IFN-gamma R level in chronic HIV infected patients was significant higher than those in HIV-negative controls (*p* < 0.01).

### The protein levels of IFN receptors and related genes during acute HIV-1 infection

**Fig. 2:**
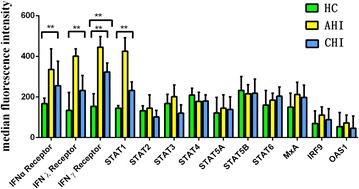
The mean fluorescence intensity (MFI) of IFNs related proteins in HIV-negative controls (HC), acute HIV-1-infected (AHI) patients and chronic HIV-1-infected (CHI) patients. The flow cytometry was used to evaluate the protein levels. All expression analysis was performed by flow cytometry using BD FACSCanto™ II with Diva software. The results are shown mean ± standard deviation, the *asterisks* denote *p* < 0.01

In the study, the following protein levels were evaluated by flow cytometry: IFN-alpha/beta R2, IFN gamma R1, IFN-lambdas R1, STAT1, STAT2, STAT3, STAT4, STAT5a, STAT5b, STAT6, IRF9, MxA, OAS1. It can be seen from Fig. 2 that the mean fluorescence intensity (MFI) of IFN-gamma R, IFN-lambdas R, IFN-alpha R and STAT1 in acute HIV infected patients were significant higher than those in HIV-negative controls (*p* < 0.01). The IFN-gamma R level in chronic HIV infected patients was significant higher than those in HIV-negative controls (*p* < 0.01).

### The correlation between CD4^+^ T cells, viral loads and mRNA levels of IFN receptors in HIV-1-infected patients

**Fig. 3:**
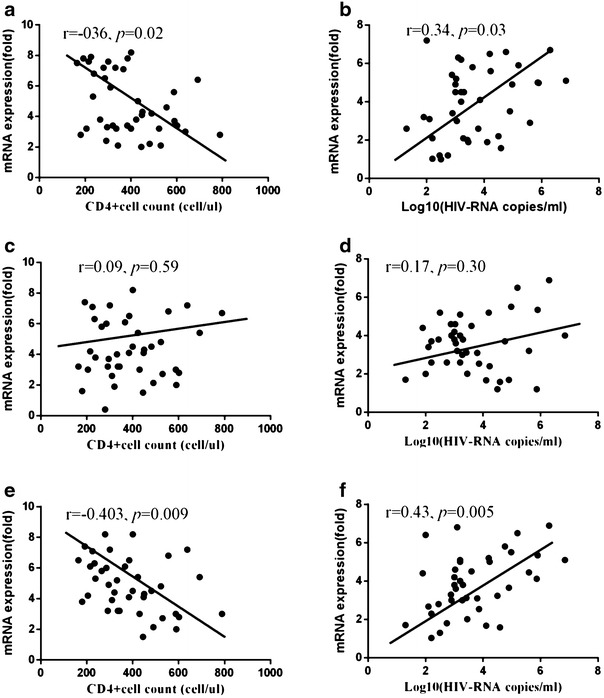
Correlation between the CD4^+^ T cells and mRNA levels of IFN-alpha receptor (**a**), IFN-gamma receptor (**c**), and IFN-lambdas receptor (**e**). Correlation between the viral loads and mRNA levels of IFN-alpha receptor (**b**), IFN-gamma receptor (**d**), and IFN-lambdas receptor (**f**). The results were performed Spearman’s rank correlation, where coefficients “r” and corresponding *p* values are indicated on each panel

The correlation between IFNs receptors and CD4^+^ T cell counts and viral loads was evaluated (Fig. 3). IFN-alpha R mRNA levels are inversely correlated with CD4^+^ T cell counts (Fig. 3a), but are positively correlated with viral loads (Fig. 3b). IFN- lambdas R mRNA levels are inversely correlated with CD4^+^ T cell counts (Fig. 3e) whereas positively correlated with viral loads (Fig. 3f).

## Discussion

IFN-lambdas are newly classified type III interferons, which use JAK-STAT pathway. Currently, the dynamics of IFN-lambdas related genes and proteins expression in the signaling pathway have not been well elaborated, especially in acute HIV-1-infected patients. In the study, we found that in acute HIV-1-infected patients, IFN- alpha R, IFN-gamma R, IFN-lambdas R, STAT1 mRNA and protein levels increased (p < 0.01), in addition, Mx1 mRNA levels in AHI are higher than those in HIV-negative subjects. In chronic HIV infected patients, IFN-gamma R levels were significant higher than those in HIV-negative controls (p < 0.01). IFN-lambdas R and IFN-alpha R mRNA levels are positively correlated with viral loads, whereas negatively correlated with CD4^+^ T-cell numbers.

Previous studies have showed that type I and III IFNs can activate similar intracellular signaling pathway and biological activities [17, 18]. In our study, IFN-alpha receptor and IFN-lambdas receptor production were up-regulated in acute HIV-1 infection. Stacey et al. [8, 9] reported that throughout the acute stage of HIV-1 infection, IFN-gamma receptor levels in infected adults steadily increase, with a peak about 20–24 days post-infection. In chronic non-progression or progression HIV-1-infected patients, IFN-gamma receptor persistently elevated. In the study, we found that the IFN-gamma R levels in both acute and chronic HIV infected patients were significant higher than those in HIV-negative controls (p < 0.01), our findings were consistent with previous study. The difference between IFN-lambdas and IFN-gamma expression may be caused by different receptor systems which activate different transcription factors and IFN regulatory factors [19]. Viral infection induced a number of genes and proteins in the signaling pathway upon viral infections, we found that Mx1 and STAT1 mRNA levels can be induced in acute HIV-1 infection. In the study, Mx1 mRNA levels are significantly higher than those of HIV-negative controls, but the MxA protein levels are similar between two groups. Several factors are responsible for the differences. Firstly, due to the deletion of three exons or the presence of a nonsense mutation, Mx1 gene becomes inactive and the translation is terminated in most mouse strains [20]. Secondly, the MxA protein will be hydrolyzed rapidly after its secretion in vitro, so there are fewer MxA protein left in cells. It has been reported that STAT1 protein can be up-regulated by IFN-alpha, gamma and lambdas in human hepatoma cells, melanoma cell lines and Raji cells, respectively [21–23].

In the study, the gene and protein expression target on PBMC rather than CD4^+^ T cells, DCs or other immune cells. In the further studies, we should sort cells and analyze the gene and protein expression on specific cell type. IFN-lambdas was discovered only a decade ago and showed great potentials in antiviral therapy with much fewer side effects because of limited receptor distribution [24, 25]. In the study, we demonstrated the dynamics of IFN lambdas and the related genes and proteins in JAK-STAT pathway in acute and chronic HIV-1 infection, and we analyzed their association with CD4^+^ T-cell counts and HIV-1 viral loads. The dynamic changes of IFNs related genes in JAK-STAT pathway in acute HIV-1 infection will deepen our understanding of the roles of IFN-lambdas in HIV pathogenesis, and our findings may provide valuable information on IFN-lambdas potential applications in clinical settings.
